# Vitamin B12 deficiency and neuropsychiatric symptoms in Lebanon: A cross-sectional study of vegans, vegetarians, and omnivores

**DOI:** 10.1371/journal.pone.0297976

**Published:** 2024-04-17

**Authors:** Omar Al Jassem, Karim Kheir, Ali Ismail, Linda Abou-Abbas, Alaa Masri, Celine Haddad, Jad El Masry, Khalil Nasrallah

**Affiliations:** 1 Faculty of Medical Sciences, Neuroscience Research Center, Lebanese University, Beirut, Lebanon; 2 Faculty of Medical Sciences, Lebanese University, Beirut, Lebanon; 3 INSPECT-LB, Institut National de Santé Publique, Epidémiologie Clinique et Toxicologie, Faculty of Public Health, Lebanese University, Fanar, Lebanon; 4 Department of Anatomy, Cell Biology, and Physiological Sciences, Faculty of Medicine, American University of Beirut, Beirut, Lebanon; Research, Training and Management International, BANGLADESH

## Abstract

**Background:**

Vitamin B_12_ deficiency is responsible for a variety of complications, particularly neurological/neuropsychiatric complications, including depression, irritability, paresthesia and insomnia. Since vitamin B_12_ is found in animal-derived products, vegans/vegetarians are at a greater risk for developing vitamin B_12_ deficiency.

**Aims:**

This study aims to investigate the occurrence of vitamin B12 deficiency among a sample of adult Lebanese population, with a particular emphasis on assessing the severity of its neurological/neuropsychiatric signs and symptoms, especially among vegans/vegetarians.

**Methodology:**

A cross-sectional study was conducted among a sample of 483 Lebanese adults. Data was collected through a standardized questionnaire that included socio-demographic characteristics, the Patient Health Questionnaire-9 (PHQ-9), Generalized anxiety disorders-7 (GAD-7), and the Insomnia Severity Index (ISI) scales.

**Results:**

Among the participants, 11.4% were in the vegan/vegetarian group, and about 43.1% had vitamin B_12_ deficiency. After analyzing the PHQ-9, GAD-7 and ISI total scores, higher scores were reported in participants with vitamin B12 deficiency, compared to individuals with normal vitamin B_12_ serum levels (p < 0.001). Regarding the diet type, vegans/vegetarians were more susceptible to developing depression compared to omnivores (mean scores of 11.92 vs 8.02 on the PHQ-9 scale, respectively, with p < 0.001). Of the patients with vitamin B_12_ deficiency, 81.1% reported having paresthesia compared to 43.7% of individuals with no vitamin B_12_ deficiency (p < 0.001).

**Conclusion:**

Vitamin B12 deficiency in Lebanon is notably high and is linked to an increased risk of developing depression, generalized anxiety disorder, insomnia, and paresthesia. Vegans/vegetarians exhibit a higher susceptibility to developing depression compared to omnivores, whereas the risk of developing insomnia, generalized anxiety disorder and paresthesia was statistically insignificant when comparing vegans/vegetarians to omnivores.

## Introduction

Vitamin B_12_, also known as cobalamin, is one of the eight B vitamins [1]. The only natural source of vitamin B_12_ in the human diet is animal-based food, like meat (2–5 g/100 g), fish (2–8 g/100 g), milk (1.5 g/100 ml), cheese (1–2 g/100 g), and eggs (2 g/100 g) [2]. Vitamin B_12_ is crucial for the maintenance of the myelin sheath and used in neurotransmitters synthesis [3]. Thus, vitamin B_12_ deficiency reflects a serious health problem that could lead to a wide variety of neurological signs and symptoms, including numbness and tingling of the limbs, decreased sensation, difficulties in walking, loss of memory, dementia, and depression [4].

Moreover, the demyelination of peripheral and central nerves, due to vitamin B_12_ deficiency, results in nerve damage and neuropsychiatric abnormalities [5]. Another important complication of vitamin B_12_ deficiency that is commonly reported is anemia [6]. Inadequate intake, malabsorption, chemical inactivation, or genetic impairment of either vitamin B_12_ transport in the blood or intracellular metabolism are the main causes of vitamin B_12_ deficiency [7].

Furthermore, a patient is said to have a vitamin B_12_ deficiency if the corresponding serum level of vitamin B_12_ is less than 150 pg/ml (111 pmol/L). However, clinicians must be vigilant when analyzing these laboratory results, since serum levels of vitamin B_12_ may be falsely increased in patients with alcoholism, a history of liver disease, or cancer [8, 9].

Vitamin B_12_ deficiency is a significant concern among individuals following vegan or vegetarian diets, as vitamin B_12_ is primarily sourced from animal-based products. The recommended daily intake for vitamin B_12_ in adults aged 18 years and above is 2.4 micrograms [10]. To meet this requirement, individuals adhering to a vegan/vegetarian diet must consider supplementation or the consumption of B_12_ -fortified foods.

Vegetarianism can be classified into different plant-based subgroups: lacto-ovo-vegetarianism (LOV) which forbids the consumption of animal flesh but permits the consumption of eggs and dairy products, ovo-vegetarianism, which is similar to LOV but forbids the consumption of dairy products, and lacto-vegetarianism, which is similar to LOV but forbids the consumption of eggs. Another type of diet is veganism, which excludes all animal-derived products [11].

Previous studies have assessed the prevalence of vitamin B_12_ deficiency in USA [12], UK [13], India [14], but no similar studies have been conducted among the Lebanese population. Hence, the present study is the first cross-sectional study done in Lebanon to assess the occurrence of vitamin B_12_ deficiency and the severity of the resulting neurological symptoms in a sample of Lebanese adults, as well as to compare these symptoms to the ones seen in the vegan/vegetarian population. Of particular interest, this study comes handy during these times, since it took over in Lebanon at a time when the country was facing one of the worst economic crises since 1850, leading to significant inflation and a great decline in the purchasing power [15]. This has resulted in considerable dietary modifications, particularly, less consumption of animal-derived products among the Lebanese population. Consequently, we hypothesized that these dietary modifications might have resulted in an increase in the occurrence of vitamin B_12_ deficiency.

In view of that, this study aims to investigate the occurrence of vitamin B12 deficiency among a sample of adult Lebanese population, with a particular emphasis on assessing the severity of its neurological/neuropsychiatric signs and symptoms, especially among vegans/vegetarians. We hypothesize that there is a significant difference in the occurrence and severity of vitamin B12 deficiency-related neurological/neuropsychiatric signs and symptoms among different dietary groups within the adult Lebanese population. Specifically, we expect a higher prevalence and greater severity of these symptoms among vegans/vegetarians compared to omnivores.

## Materials and methods

### Study design

The study design is a cross-sectional web-based survey, which was conducted in Lebanon during the period from April to June 2023. The survey was administered online through a Google form and was recruited through social media platforms using the snowball sampling technique.

### Participants

Participants in this study included individuals from all the eight governorates in Lebanon. Participants were eligible if they were older than 18 years of age, residing in Lebanon. People who did not consent to this study after being notified about the privacy and confidentiality of the data collection process, as well as individuals who suffer from psychiatric illnesses including depression, were excluded.

### Ethical considerations

The study was conducted after receiving approval from the neuroscience research center, Faculty of Medical Sciences, Lebanese University (ID 181/2/2023).

### Procedure

Prior to participating in the web-based survey, participants were presented with a consent form on the first page. This form provided detailed information about the study’s purpose, scope, data usage, privacy measures, and data management procedures. Participants were required to read and agree to the terms outlined in the consent form, indicating their voluntary participation and understanding of the study before proceeding further. Participants were assured of the confidentiality, privacy, and anonymity of their information. Furthermore, participants were under no obligation and had the freedom to withdraw from the study at any point. Consent was obtained through a Yes/No question stating: ‘Do you agree to participate in the study?’ All collected data are confidential and are only accessible by the team for research purposes.

### Sample size and power

Using “The Survey System” online calculator (surveysystem.com) and selecting 95% confidence interval, and 5% margin of error, we obtained at least the number 382 as a sample size for this study. Post hoc power analysis using G power (version 3.1.9.2) indicated that sample of a subset (n  =  209) was sufficient to achieve the power of the study  =  0.82 indicating a strong ability to detect the specified effect size with chi-squared goodness-of-fit test for contingency tables at 0.05 level of alpha, an effect size (w) of 0.20, and 1 degree of freedom (df).

### Data collection methods, instruments, and measurement

The data collection process started on the 17 April of 2023, and ended on the 26 June of 2023. Data collection was accomplished using a questionnaire allows the assessment of several parameters, including the sociodemographic characteristics of the participants (age, gender, highest level of education, Marital status, employment status) the presence or absence of vitamin B_12_ deficiency based on the last blood tests if available, the type of diet adopted during the time of the study conduction, and finally, the assessment of neurological/psychological manifestations, such as depression, using the PHQ-9 scoring system, generalized anxiety disorder (GAD) using the GAD-7 scale, insomnia, using the ISI as a screening instrument, as well as paresthesia.

While the severity of depression, GAD, and insomnia, if present, was assessed based on reputable and reliable scoring systems, paresthesia was only assessed based on the subjective reports of each patient, and no tool or scoring instrument was followed to measure the intensity of paresthesia.

### PHQ-9 scoring system

The PHQ-9 scoring system is a 9-item self-report questionnaire designed to assess depression. The total scores range between 0 and 27. The depression severity is considered to be: none-minimal for a score of 0–4, mild for a score of 5–9, moderate for a score of 10–14, moderately severe for a score of 15–19, and severe for a score of 20–27 [16]. Furthermore, major depressive disorder is considered to be present if of the 9 items of the PHQ-9 scoring system, 5 or more are checked as at least ’more than half the days’, and/or if either item 1 or 2 is checked as at least ’more than half the days’ [17].

### Generalized anxiety disorder 7-Item scale (GAD-7)

The GAD-7 is a self-reported questionnaire for screening and measuring the severity of GAD. It has seven items, which measure the severity of various signs of GAD according to reported response categories with assigned points that range from 0 (not at all sure) to 3 (nearly every day). The total score is the sum of all items, with higher values representing greater severity of symptoms. The GAD-7 scores range from 0 to 21. The severity of GAD is considered to be: absent or low risk for a score of 0–4, mild for a score of 5–9, moderate for a score of 10–14, and severe for a score ≥ 15 [18].

### Insomnia severity index (ISI)

The ISI is a brief tool that aims to screen for the presence and severity of insomnia. According to the insomnia severity index (ISI), a patient is considered to have no clinically significant insomnia for a score of 0–7, subthreshold insomnia for a score of 8–14, clinical insomnia of moderate severity for a score of 15–21, and severe clinical insomnia for a score of 22–28 [19].

### Statistical analysis

The collected data was entered into Microsoft Excel and analyzed using the Statistical Package of the Social Sciences (SPSS) v.25. Descriptive statistics were conducted where continuous variables were expressed as mean ± standard deviation (SD), while categorical variables were presented as numbers and percentages. Concerning the bivariate analysis, the Student’s t-test was presented for the continuous variables if the distribution is normal, while the Mann-Whitney U test was presented if the distribution is skewed. Moreover, for the categorical variables, the Chi-square test was used. For multivariable analysis, the logistic regression was used. All the variables with a p-value below 0.2 were entered in the multivariable logistic regression. Adjusted odds ratios with 95% confidence intervals were reported. A p-value < 0.05 is considered to be statistically significant.

## Results

Table 1 summarizes the characteristics of 483 participants in the study. Among them, 72.3% were females. The mean age was 27.25 and a standard deviation of 11.9 years. The majority of the participants (90.9%) had a university education level. Within the sample, 11.4% identified as vegetarians, and 29.2% reported taking vitamin B12 supplements.

**Table 1:** Baseline characteristics of the study participants (N = 483).

Characteristics	Total (N = 483)	%
**Age (years) Mean (SD)**	27.25 (11.9)	
**Gender n (%)**		
Male	134	27.7
Female	349	72.3
**Education level n (%)**		
Primary school	10	2.1
Secondary school	34	7
University	439	90.9
**Marital status n (%)**		
Married	102	21.1
Single	381	78.9
**Employment status n (%)**		
Full time	102	21.1
Part time	85	17.6
Unemployed	275	56.9
Retired	21	4.3
**Place of residence n (%)**		
North Lebanon	141	29.2
Mount Lebanon	126	26.1
Akkar	52	10.8
Bekaa	44	9.1
Beirut	42	8.7
South Lebanon	34	7
Nabatieh	26	5.4
Baalbeck-Hermel	18	3.7
**Type of diet n (%)**		
Omnivore	428	88.6
Vegan/vegetarian	55	11.4
**Duration adopting vegan/vegetarian diet n (%)**		
Less than 1 year	5	9.09
Between 1 and 5 years	28	50.9
More than 5 years	22	40
**Taking vitamin B**_**12**_ **supplements n (%)**		
Yes	141	29.2
No	342	70.8

n: number, %: percentage, SD: standard deviation.

Among the 483 individuals, 274 (56.7%) were never tested for Vitamin B12 deficiency, 90 (18.6%) were found to have vitamin B12 deficiency, and 119 (24.6%) exhibited normal Vitamin B12 levels based on their last lab test results. Consequently, 43.1% of our group was identified with Vitamin B12 deficiency.

In Table 2, a comparison between healthy individuals and those with vitamin B12 deficiency is presented. The mean age for participants with vitamin B12 deficiency was 29.6 ± 13.94, while for those without deficiency; it was 27.63 ± 11.5, yielding a p-value of 0.281. Among the 209 individuals, 22 (40.7%) with vitamin B12 deficiency were male, and 32 (59.3%) had normal serum levels of vitamin B12, resulting in a p-value of 0.740. The percentage of vitamin B12 deficiency among vegan/vegetarian participants was 54.05%, compared to 40.7% in omnivorous patients, with a p-value of 0.147, indicating a statistically non-significant difference.

**Table 2:** Comparison of characteristics between healthy individuals and those with vitamin B12 deficiency (N = 209).

	Vitamin B_12_ deficiency				
	Yes (n = 90)	%	No (n = 119)	%	P-value
**Age (years) Mean (SD)**	29.6 (13.94)	-	27.6 (11.57)	-	0.281
**Gender**					
Male	22	40.7	32	59.3	0.751
Female	68	43.9	87	56.1	
**Educational level**					
Primary school	5	100	0	0	0.035*
Secondary school	8	40	12	60	
University	77	41.8	107	58.2	
**Social status**					
Married	22	45.8	26	54.2	0.740
Single	68	42.	93	57.8	
**Employment status**					
Employed	32	38.1	52	61.9	0.480
Full time	18	36	32	64	0.654
Part time	14	41.2	20	58.8	
Unemployed	53	46.9	60	53.1	
Retired	5	41.7	7	58.	
**Type of diet**					
Omnivore	70	40.7	102	59.3	0.147
Vegan/vegetarian	20	54.1	17	45.9	
**Duration adopting vegan/vegetarian diet**					
Less than 1 year	3	75	1	25	0.640
Between 1 and 5 years	11	55	9	45	
More than 5 years	6	46.2	7	53.8	
**Taking Vitamin B**_**12**_ **supplements n**					
Yes	44	32.8	90	67.2	<0.001*
No	46	61.3	29	38.7	

n: number, %: percentage, SD: standard deviation, *P-value less than 0.05 is considered significant.

Among individuals taking vitamin B12 supplements (N = 134), 44 (32.8%) had vitamin B12 deficiency, whereas 46 (61.3%) individuals who did not take vitamin B12 supplements (N = 75) exhibited deficiency, resulting in a highly significant p-value of < 0.001. This finding underscores a statistically significant association between vitamin B12 deficiency and supplement usage.

In Table 3, it is evident that individuals with vitamin B12 deficiency exhibited significantly higher total scores for PHQ-9 (11.92 ± 6.16), GAD-7 (10.02 ± 6.97), and ISI (10.92 ± 5.44) compared to those with normal serum levels of vitamin B12 (8.02 ± 6.06, 5.73 ± 5.42, and 7.58 ± 5.75, respectively). The observed differences were statistically significant (p-value < 0.001).

**Table 3:** Total scores of PHQ-9, GAD-7, and ISI based on vitamin B12 lab test status.

		Vitamin B_12_ deficiency		
	Total (n = 483)	Yes (n = 90)	No (n = 119)	P-value
**PHQ-9 Mean (SD)**	9.88 (6.25)	11.92 (6.16)	8.02 (6.06)	<0.001*
**GAD-7 Mean (SD)**	9.11(5.73)	10.02 (6.97)	5.73 (5.42)	<0.001*
**ISI Mean (SD)**	6.72 (6.16)	10.92 (5.44)	7.58 (5.75)	<0.001*

PHQ-9: Patient Heath Questionnaire-9, GAD-7: General Anxiety Disorder-7, ISI: Insomnia Severity Index, SD: Standard Deviation, *P-value less than 0.05 is considered significant.

**Fig 1:**
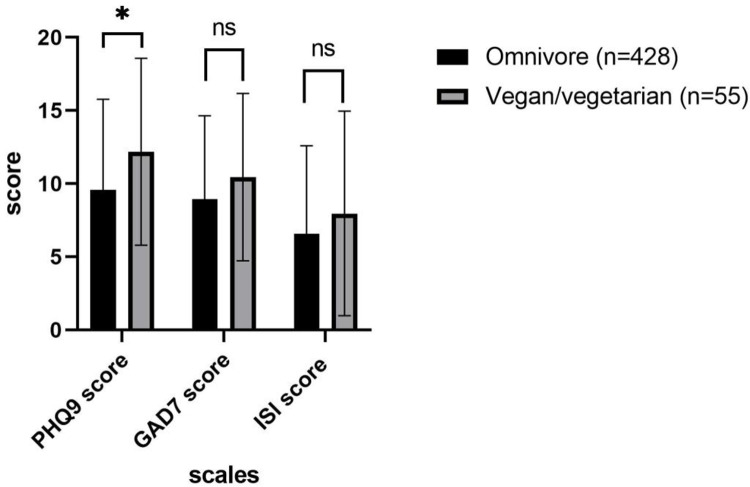
Mean and standard deviation of PHQ-9, GAD-7, and ISI total scores based on diet type. *P-value less than 0.05 is considered significant. Ns: statistically non-significant.

The mean PHQ-9 total scores were notably higher in the vegan/vegetarian population (12.18) compared to omnivorous participants (9.59), yielding a significant p-value of <0.005. Conversely, no significant differences in GAD-7 and ISI total scores were observed between the two groups (10.45 vs. 8.94 for GAD-7 and 7.96 vs. 6.56 for ISI in vegans/vegetarians and omnivorous individuals, respectively) (Fig 1).

Referring to Table 4 the percentage of paresthesia was significantly higher in females (60.7%) than males (44%), with a p-value of 0.001. Furthermore, this percentage was significantly higher in individuals with vitamin B_12_ deficiency (81.1%), compared to individuals with normal serum levels of vitamin B_12_ (43.7%), resulting in a significant p-value < 0.001. However, no association was found between paresthesia and the diet type: the percentage of paresthesia in omnivores was 56.5% compared to 52.7% in vegans/vegetarians (p-value = 0.346).

**Table 4:** Distribution of paresthesia according to gender, vitamin B_12_ deficiency and diet type.

	Paresthesia				
	Yes	%	No	%	P-value
**Gender n (%)**					
Female	212	60.7	137	39.3	0.001*
Male	59	44	75	56	
**Vitamin B**_**12**_ **deficiency n (%)**					
Yes	73	81.1	17	18.9	<0.001*
No	52	43.7	67	56.3	
**Diet type n (%)**					
Omnivorous	242	56.5	186	43.5	0.346
Vegan/vegetarian	29	52.7	26	47.3	

n: number, %: percentage

*P-value less than 0.05 is considered significant.

Multivariable logistic regression analysis showed that age, gender, marital status, type of diet, and employment status were not significantly associated with vitamin B_12_ deficiency Table 5.

**Table 5:** Multivariable logistic regression of the factors associated with vitamin B12 deficiency.

Variables		OR	95% CI	p-value
**Age**		0.955	0.935–4.223	0.054
**Gender**	Male	1.00		
	Female	0.807	0.390–1.668	0.563
**Marital status**	Married	1.00		
	single	0.588	0.198–1.742	0.338
**Type of diet**	Omnivorous	1.00		
	Vegan/vegetarian	0.503	0.273–1.069	0.074
**Employment status**	Employed	1.00		
	Unemployed	0.524	0.270–1.017	0.056
	Retired	2.021	0.353–11.567	0.429

SE: Standard Error, OR: odds Ratio, CI: confidence Interval

*P-value less than 0.05 is considered significant.

## Discussion

The present study was conducted to explore the occurrence of vitamin B12 deficiency among a sample of adult Lebanese population, with a particular emphasis on assessing the severity of its neurological/neuropsychiatric signs and symptoms, especially among vegans/vegetarians. Our findings showed a vitamin B12 deficiency of 43.1% in our sample. While our initial hypothesis proposed a significant difference in the occurrence and severity of vitamin B12 deficiency-related neurological/neuropsychiatric symptoms among different dietary groups, our study results did not fully support this hypothesis. No significant difference in deficiency rates between vegans/vegetarians (54.1%) and omnivores (40.7%) was found suggesting that the deficiency was not strongly linked to diet.

Higher scores for depression, generalized anxiety disorder and insomnia were detected in patients with vitamin B_12_ deficiency compared to patients with normal vitamin B12 serum levels. However, when comparing vegans/vegetarians to omnivores, only depression was significantly more severe in the vegan/vegetarian group.

Similarly, paresthesia was significantly more common in patients with vitamin B_12_ deficiency, but no association was found between the presence of paresthesia and the vegan/vegetarian diet.

### PHQ-9, GAD-7 and ISI total scores according to vitamin B_12_ lab test

All total scores related to depression, generalized anxiety disorder and insomnia were significantly higher among participants with vitamin B_12_ deficiency (p < 0.001 for all three variables).

The mean score of PHQ-9 in individuals with vitamin B12 deficiency was 11.92, indicating moderate depression, compared to 8.02 in participants without vitamin B_12_ deficiency, suggesting only mild depression in these individuals, according to the PHQ-9 scale.

These results suggest that low serum levels of vitamin B_12_ are associated with a higher severity of depression. Our findings are congruent with the results of several studies, including a Chinese study [20] that found that vitamin B_12_ deficiency was significantly related to the development of depressive symptoms, as well as a Korean study [21] that demonstrated an association between low serum levels of vitamin B_12_ and a greater incidence of depression during follow-up.

Additionally, participants with vitamin B_12_ deficiency were found to have higher GAD-7 total scores compared to participants without vitamin B_12_ deficiency (10.02 vs 5.73, respectively), suggesting moderate versus mild anxiety levels in participants with vitamin B_12_ deficiency versus individuals with normal serum levels of vitamin B_12_, respectively.

This association between vitamin B_12_ deficiency and greater severity of GAD, as shown by the present study, is consistent with the findings of several studies. In fact, a recent Chinese study [22] that has been conducted using the same scale as the one used in our study (GAD-7 scale), found that lower serum levels of vitamin B_12_ were associated with greater risk of severe anxiety. However, a cross-sectional study conducted in India [23] found no direct correlation between vitamin B_12_ deficiency and GAD, but a considerable higher risk of developing GAD in individuals with hyperhomocysteinemia. Hence, further studies are needed to clarify whether vitamin B_12_ deficiency is directly associated with a higher prevalence and severity of GAD.

Moreover, participants with vitamin B_12_ deficiency were found to have higher ISI total scores than participants without vitamin B_12_ deficiency (10.92 vs 7.58, respectively), indicating sub-threshold insomnia in the former group, compared to non-clinically significant insomnia in the latter group.

Although this study demonstrated that low serum levels of vitamin B_12_ were associated with increased severity of insomnia, our results do not match with the findings of other studies. A study conducted in 2019 [22] found that vitamin B_12_ levels did not significantly affect the severity of insomnia. Furthermore, it is of particular interest to mention than another study [24] found that vitamin B_12_ levels were considerably higher in insomniac individuals, which does not correlate with our findings. Consequently, additional studies are needed to investigate the relation between cobalamin levels and insomnia.

### PHQ-9, GAD-7and ISI total scores according to diet type

Based on our study findings, vegans/vegetarians had a mean total score of 12.18 according to the PHQ-9 scale, which correlates with moderate depression, compared to mild depression (mean total score of 9.59) in participants following an omnivorous diet. As a result, the severity of depression was significantly higher in the vegan/vegetarian group (p-value = 0.004). These findings are similar to the results mentioned in the literature. Of note, a recent meta-analysis [25] concluded that vegetarians are at greater risk of developing depression compared to omnivores, even though no significant differences in the mean score of depression were detected between the two groups. Similarly, another systemic review and meta-analysis [26] showed that following a vegan/vegetarian diet was associated with a higher risk of depression.

In contrast, no significant association was detected between vegan/vegetarian diet and the development of GAD or insomnia, when compared to individuals following an omnivorous diet (p values of 0.066 for GAD and 0.113 for insomnia). These results are aligned with the findings of a French study [27] that found no relation between plant-based diet and anxiety. However, a systemic review and meta-analysis [26] showed that lower scores of anxiety were detected in participants adopting a vegan/vegetarian diet. In addition, when assessing the relation between vegan/vegetarian diet and insomnia, a study about sleep disorders [28] concluded that insomnia was more frequent among omnivores, compared to individuals following a vegan/vegetarian diet.

### Paresthesia

It is of particular interest to mention that female participants were more likely to report the presence of paresthesia compared to males (60.7% vs 44%, respectively), resulting in a significant p-value (p < 0.001). No significant association was found between the paresthesia and the diet type (56,5% in omnivores vs 52,7% in vegans/vegetarians), according to our study. Nevertheless, a recent study [29] showed that the frequency of paresthesia was significantly higher in vegan/vegetarian participants compared to omnivores (p = 0,04).

Although paresthesia was not considerably affected by the diet type according to our study, it was significantly influenced by the serum levels of vitamin B_12_: paresthesia was significantly higher in individuals with vitamin B_12_ deficiency (81,1%) compared to individuals with normal serum levels of vitamin B_12_ (43 7%), with a p-value < 0.001.

### Comparison of vitamin B12 deficiency between vegans/vegetarians and omnivores

The difference in serum concentrations of vitamin B_12_ between vegans/vegetarians and omnivores was not statistically significant; a result that is not compatible with the findings of another study [29] that found that serum concentrations of vitamin B_12_ were significantly lower in the vegetarian group compared to omnivores. The lack of a significant difference in serum cobalamin levels of vegans/vegetarians compared to omnivores, as shown in our study, could be due to the fact that Lebanon is currently facing one of the world’s worst economic crises. Consequently, dietary modifications were observed in a large portion of omnivorous patients, who reported eating much less meat, poultry, fish and animal-derived products, all being essential sources of vitamin B_12_, during the economic crisis, which has led to a decrease in the serum levels of vitamin B_12_ in omnivores too. This could also explain why vitamin B_12_ deficiency among our sample of Lebanese adults exceeded the prevalence of cobalamin deficiency in many countries around the world. In our study, vitamin B12 deficiency among the Lebanese adults was as high as 43.1% compared to only 4.8% in France [30], 8% in Liechtenstein [31], 14% in Australia [32] and 14.9% in South India [33].

### Strengths and limitations

In addition to being the first study to compare vitamin B12 deficiency between vegans/vegetarians and omnivores, this research benefits from the utilization of rigorously validated scales. These validated tools ensure the reliability and accuracy of the gathered data, enhancing the robustness of our findings. Moreover, the comprehensive nature of this study, examining not only the occurrence of vitamin B12 deficiency, but also its impact on mental health and the specific dietary practices of vegans/vegetarians, highlights this critical health issue in Lebanon. However, our study still has several limitations. The study’s sampling strategy, with a predominance of university students, raises concerns about potential selection bias, which can compromise the study’s ability to provide a precise representation of vitamin B12 deficiency in the elderly. Hence, additional studies to assess the neuropsychological complications of vitamin B12 deficiency among the elderly population in Lebanon are needed. Additionally, the utilized self-report scales might have provoked inherent limitations. Response bias, characterized by consistent or socially desirable patterns, can impact the reliability of the collected data. Memory recall issues, particularly with distant events, might have resulted in errors. Furthermore, the relatively small sample size of vegans/vegetarians (only 55) might have hindered the detection of significant differences in vitamin B12 deficiency rates between different dietary groups. In addition, the subjective nature of paresthesia, and the lack of standardized scales to confirm its presence and assess its severity, introduces a potential measurement bias. As cross-sectional studies can have limitations in establishing causal relationships, more comprehensive longitudinal studies or meta-analyses with larger samples of vegans/vegetarians could be of great benefit.

## Conclusion

Vitamin B_12_ deficiency is common among the Lebanese population, affecting about 43.1% of the general population. This high prevalence might be associated with the current economic crisis, and the reduced consumption of animal-based products due to financial difficulties. The study’s findings underscore the significant impact of vitamin B12 deficiency on psychological well-being, highlighting the need for targeted interventions in Lebanon. Health policymakers should prioritize public awareness campaigns to educate the population about B12 deficiency and its implications for mental health. Healthcare providers must receive training to recognize deficiency symptoms, and regular screenings should be integrated into healthcare protocols, particularly for vulnerable groups like vegans and vegetarians. Policy support for fortifying commonly consumed foods with B12 is essential, and mental health programs should include B12 level assessments. Further research and collaboration with educational institutions are crucial to comprehensively address this issue and enhance the overall mental health landscape in Lebanon. Additional studies are also needed to investigate other neurological complications associated with vitamin B_12_ deficiency.

## Supporting information

S1 File Study data. All patient results and demographics information.
(XLSX)

S1 Data (DOCX)
