# Correction: Vitamin B12 deficiency and neuropsychiatric symptoms in Lebanon: A cross-sectional study of vegans, vegetarians, and omnivores

**DOI:** 10.1371/journal.pone.0326220

**Published:** 2025-06-12

**Authors:** The PLOS One Staff

## Notice of Republication

The author order was incorrect. The correct order is the following:

Omar Al Jassem, Karim Kheir, Ali Ismail, Linda Abou-Abbas, Alaa Masri, Celine Haddad, Jad el Masri, Khalil Nasrallah.

## Supporting information

**S1 File.** Originally published, uncorrected article.

**S2 File.** Republished, corrected article.
